# Effects of pain and depression on the relationship between household solid fuel use and disability among middle-aged and older adults

**DOI:** 10.1038/s41598-022-25825-8

**Published:** 2022-12-08

**Authors:** Zhihao Jia, Yan Gao, Liangyu Zhao, Suyue Han

**Affiliations:** School of Physical Education, Shandong University, Jinan, 250061 China

**Keywords:** Health occupations, Environmental impact, Human behaviour

## Abstract

Household air pollution (HAP) is suggested to increases people's risk of disability, but mediating mechanisms between HAP and disability remains under-investigated. The aim of this study was to investigate the underlying mechanisms between household air pollution and disability in middle-aged and older adults (i.e., older than 45 years) using a nationally representative prospective cohort. In total, 3754 middle-aged and older adults were selected from the China Health and Retirement Longitudinal Study. Correlation analysis and logistic regression analysis were employed to estimate the association between HAP, pain, depression and disability. Finally, three significant mediation pathways through which HAP directly impacts disability were found: (1) pain (B = 0.09, 95% CI 0.01, 0.02), accounting for 15.25% of the total effect; (2) depression (B = 0.07, 95% CI 0.004, 0.02), accounting for 11.86% of the total effect; (3) pain and depression (B = 0.04, 95% CI 0.003, 0.01), accounting for 6.78% of the total effect. The total mediating effect was 33.89%. This study clarified that HAP can indirectly affect disability through the respective and serial mediating roles of pain and depression. These findings potentially have important implications for national strategies concerning the widespread use of clean fuels by citizens.

## Introduction

Long-term exposure to household air pollution (HAP) caused by solid fuel combustion (like wood and coal) is one of the major causes of ill health in humans^1–4^. HAP caused by solid fuel accounts for 4–4.5% of the global disease burden, endangering the health of approximately 400–700 million people^1^. Similar to ambient air pollution, HAP exerts effects on neurobehavioural and psychological outcomes^5,6^. The relevant data show that 7 million people die each year from air pollution, of which more than half (3.8 million) of these deaths are the result of HAP exposure^7,8^. In China, due to economic development and government support, an increasing number of people are able to use cleaner fuels such as solar energy, natural gas, and biogas^9^. However, nearly 50% of Chinese citizens still rely wholly or partly on solid fuels that produce large amounts of household air pollutants (wood, charcoal, manure, crop residues, and coal, as well as traditional stoves or open fires for use in cooking, lighting, and heating)^10^. Additionally, it is worth noting that older adults in China tend to be more vulnerable to HAP from solid fuel use than younger people because they (especially those in retirement) spend more time in indoor environments^11,12^. Therefore, long-term exposure to HAP has a certain degree of influence on the risk of disability in middle-aged and older adults (i.e., older than 45 years)^13–15^. This disparity in age is more pronounced in China with the degree of aging is gradually deepening ^16,17^. Therefore, it is necessary to further explore the relevant mechanisms by which HAP affects middle-aged and older adults health.

Disability is considered to be an important factor that can hinder daily life^18^. This may be the result of the interaction of various biological, physiological, and social factors. Many studies have identified that disability in activities of daily living (ADLs) is one of the main reasons for the loss of independence and the need for long-term care in elderly individuals^19,20^. In China, nearly 85 million people have disabilities that negatively affect their daily lives, and the disability rate among those over the age of 45 is increasing year by year^21^. Medical studies have shown that pain and depression can produce or exacerbate disabilities^22,23^. Importantly, these conditions that predispose one to disabilities or exacerbate disabilities are often closely related to HAP caused by solid fuel^9,24,25^.

Recently, epidemiological studies reported that long-term exposure to HAP from solid fuel use not only affects outcomes related to disability but also impacts physical and psychological outcomes, such as pain and depression. For example, the World Health Survey (WHS) linked HAP caused by the use of solid fuels to angina pectoris and increased the incidence of eye soreness and headaches in a Guatemalan population^26,27^. In addition, a controlled trial reported an association between HAP and depression in 1756 women in India and indicated that the degree of depression among women exposed to HAP was 23.4% higher than that among those not exposed to HAP^28^. A similar conclusion was drawn from a study regarding depression in the elderly population in China. Middle-aged and older adults who were chronically exposed to HAP produced by solid fuel had a 1.26–1.49 times higher risk of depression than middle-aged and older adults who were not exposed to HAP produced by solid fuel^12,29^. More importantly, a growing amount of evidence has identified that pain and depression are associated with disability^30–36^. Therefore, this study proposed the hypothesis that pain and depression may be two potential mediating mechanisms between HAP and disability.

Recently, epidemiological studies reported that long-term exposure to HAP from solid fuel use not only affects outcomes related to disability but also impacts physical and psychological outcomes, such as pain and depression.This is because household air pollutants brought about by the combustion of solid fuels, such as CO/NO_2_ and particulate matter, can trigger inflammatory responses, immune responses and even a series of oxidative stress responses^37,38^. These responses can lead to a range of respiratory, cardiovascular, and other chronic diseases in individuals. Therefore, physical pain, the most common physical manifestation of these chronic diseases^39,40^, is likely to be as affected by HAP. There is also intuitive evidence of a strong association between household air pollution exposure and pain. For example, the World Health Survey (WHS) linked HAP caused by the use of solid fuels to angina pectoris and increased the incidence of eye soreness and headaches in a Guatemalan population^26,27^. In addition, previous studies have also found that neuroinflammation and related oxidative stress caused by the inhalation of these pollutants also have an impact on people's mental health, such as depression^41,42^. More intuitive research also further proves the correlation between HAP and depression. For example, a controlled trial reported an association between HAP and depression in 1756 women in India and indicated that the degree of depression among women exposed to HAP was 23.4% higher than that among those not exposed to HAP^28^. A similar conclusion was drawn from a study regarding depression in the elderly population in China. Middle-aged and older adults who were chronically exposed to HAP produced by solid fuel had a 1.26–1.49 times higher risk of depression than middle-aged and older adults who were not exposed to HAP produced by solid fuel^12,29^. More importantly, a growing amount of evidence has identified that pain and depression are associated with disability^30–36^. Therefore, this study proposed the hypothesis that pain and depression may be two potential mediating mechanisms between HAP and disability.

Interestingly, several studies have found that pain is linked with depression^43–45^. Individuals who experience pain often report that pain negatively affects their mental function^46^. Studies have confirmed that pain could increase the risk of depression and the possibility of recurrence^47^. In addition, Hall et al. proposed and verified a mediating role of depression between pain and disability, and the study confirmed the theoretical model of psychological distress linking initial pain and long-term disability^48^. Clinical studies have also found that treating depression in patients with chronic pain is one of the main approaches that can be used to reduce their disability risk^49,50^. Thus, based on the above, it was hypothesized that pain and depression may also have serial mediating effects between HAP and disability.

Disability is very common among older people. Considering the burden of disability on individuals and society, it is imperative to identify modifiable disability risk factors due to China's ageing population and problems concerning air pollution. To the best of our knowledge, although the relationship between HAPs and disability has been suggested to be relevant, until now, no study has explored the potential link between HAP and disability through serial mediation models. Therefore, in this study, we attempted to investigate the underlying pathways in the relationship between HAP and disability among middle-aged and older adults in China. We examined the associations among HAP, pain, depression, and disability based on four waves of data from a nationally representative prospective cohort, the China Health and Retirement Longitudinal Study (CHARLS), including the baseline survey data and three periods of follow-up. Additionally, we explored the mediating roles of pain and depression in accordance with the conceptual model shown in Supplementary Fig. 1. The results provide evidence of the meditating effects of pain and depression, help guide future measures to prevent disability, and can be used as a basis for the creation of policies concerning the environment and health.

## Methods

### Data source

The CHARLS was conducted in 2011, with a follow-up every 2 years. The CHARLS is a national longitudinal cohort study that uses a multistage stratified sampling design^51^. The study protocol complied with the ethical guidelines of the 1975 Declaration of Helsinki and was approved by the Peking University Ethics Committee (approval number: IRB00001052-13074). Presently, the baseline survey data (Wave I, CHARLS 2011) and three follow-up survey datasets (Wave II, CHARLS 2013; Wave III, CHARLS 2015; Wave IV, CHARLS 2018) can be accessed through the official website of CHARLS (http://charls.pku.edu.cn/). On November 19, 2021, we applied online for permission to use the CHARLS database and collated the data after obtaining approval.

**Figure 1:**
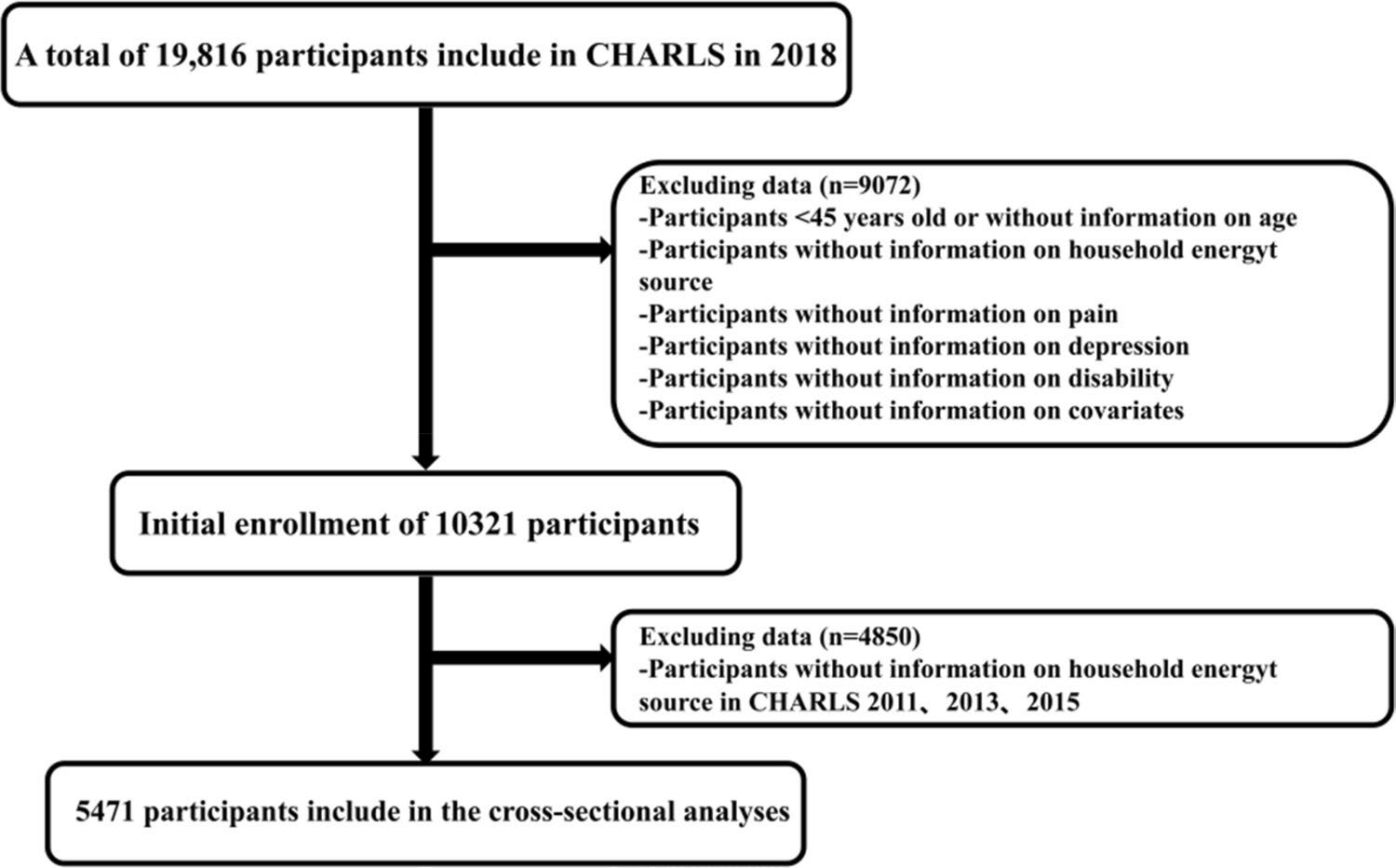
Inclusion process of participants (N = 5471).

We used participants from the 2018 CHARLS as our study population (n = 19,816). Middle-aged and older adults aged 45 years and above were included as study participants. The exclusion criteria for this study were as follows: (1) adults aged 45 years or younger who were included in the 2018 CHARLS (n = 424); (2) those who did not provide information on sex, age, residence, hukou, education level, marital status, income level, smoking status, drinking status, and chronic disease status in the 2018 CHARLS (n = 5279); and (3) those who did not provide information on HAP, pain, depression, and disability in the 2018 CHARLS (n = 3793). Ultimately, 8320 participants were initially included. Our study aimed to find some evidence for the relationship between long-term exposure to HAPs and negative health outcomes. If we only relied on the questionnaire items on HAP in the CHARLS 2018, we could measure whether there was HAP exposure in the short term but not the long term. Therefore, we performed further screening. We selected only the participants in 2018 who participated in the previous surveys (2011 CHARLS, 2013 CHARLS and 2015 CHARLS) and responded to household air pollution-related questionnaire items as our final study participants (n = 5437) (Fig. 1).

### Use of solid fuels

In the CHARLS, the household energy sources come from the Housing Characteristics section. According to the CHARLS, the household energy sources were collected through a structured questionnaire asking the participants what type of fuel they used to cook or heat^52^. Specifically, participants were asked to provide specific information about the main heating energy used in their home (what is the main heating energy source?) and the main source of cooking fuel (what is the main source of cooking fuel?). The heating energy was divided into (1) solar; (2) coal; (3) natural gas; (4) liquefied petroleum gas; (5) electric; (6) crop residue/wood burning; and (7) concentration heating. Cooking fuel sources were divided into (1) solar; (2) coal; (3) natural gas; (4) liquefied petroleum gas; (5) electric; and (6) crop residue/wood burning. Of the energy sources reported above, crop residue/wood burning and coal are defined as solid fuels, while solar, natural gas, liquefied petroleum gas, electric and concentration heating are defined as clean fuels because they produce less air pollution than solid fuels^11^. Based on previous studies, we considered the use of solid fuel and its duration as surrogates of HAP^52^. Referring to the studies of Cao Limin et al.^14^, Liu Yuhong et al.^52^ and after adjustment for the purpose of our study, we defined the duration of solid fuel use by dividing it into three levels based on the type of fuel used for cooking and heating reported at each follow-up visit. If an individual reported consistently using clean fuels for cooking or heating and never using solid fuels for cooking or heating at the time of the four surveys, the participant was considered free of HAP exposure (0 years) and received a score of 1; if an individual reported cooking or heating with solid fuels at least once, but didn’t report using solid fuel cooking and heating four times in a row and received a score of 2; and if an individual reported consistently using solid fuels and never using clean fuels for cooking or heating at the time of the four surveys, the participant was considered to have sustained HAP exposure (≥ 8 years)and received a score of 3. The specific questionnaire items are shown in Supplementary Table 1.

### Disability

In our study, disability came from the Health Status and Functioning section of the 2018 CHARLS. In the CHARLS, disability was measured by ADL and IADL scales^53,54^. The ADL disability scores quantify the ability to perform seven activities, including dressing, bathing, eating, getting out of bed, using the toilet, controlling urination and defecation, and walking^55^. The IADL disability score quantifies the ability to engage in 6 tasks and determines whether the respondent can make decisions alone and interact with their surroundings. Individuals with IADL disability typically have difficulties shopping, managing money, cooking, doing chores, making phone calls, and taking medicine^55,56^. Each answer for the ADL and IADL scales was divided into four levels, and the four answer options were as follows: "No, I do not have any difficulty", 1 point; "I have difficulty but can still do it", 2 points; "I have difficulty and need help", 3 points; and "I cannot do it", 4 points. In this analysis, we refer to previous studies with ADL scores ranging from 7 to 28^57^, and IADL scores ranging from 6 to 24 points^56^. Finally, we added the IADL disability and ADL disability scores together. The higher the scores were, the more severe the disability of middle-aged and older adults^56,58^. The specific questionnaire items are shown in Supplementary Table 1.

### Pain

In our study, pain comes from the Health Status and Functioning section of 2018 CHARLS. Body pain was determined using a question in the CHARLS questionnaire. The respondents were asked "Are you often troubled with any body pains?", and the responses were coded as follows. If the respondent answered "none", it was coded as 1; "a little" was coded as 2; "somewhat" was coded as 3; "quite a bit" was coded as 4; and "Very" was coded as 5.The specific questionnaire items are in Supplementary Table 1.

### Depression

In our study, depression comes from the Health Status and Functioning section of 2018 CHARLS. Depression levels were measured using a short version of the Centre for Epidemiological Studies Depression Scale (CES-D) questionnaire, which is widely used in population-based studies^59^. The short version of the CES-D has sufficient reliability and validity in China middle-aged and older adults^60^. The short version of the CES-D consists of 10 items referring to the respondent’s feelings and behaviour during the previous week^61^. Each item was measured on a 4-point scale: 0 [rarely or none of the time (< 1 day)], 1 [some or a little of the time (1–2 days)], 2 [occasionally or a moderate amount of the time (3–4 days)], and 3 [most or all of the time (5–7 days)]. Affirmatively worded statements were reverse coded before summation (items 5 and 8). Total CES-D-10 scores could range from 0 to 30, with higher scores indicating more pronounced depressive symptoms.The specific questionnaire items are in Supplementary Table 1.

### Control variables

According to previous studies^50,62^, we used control variables, including age, sex (male or female), place of residence (rural or urban communities), household registration (nonagricultural or rural), marital status (married or single; divorced or widowed), education level (“illiterate (had not attended school)”, below primary school, junior high school, senior high school, vocational school, and junior college and above), annual household income, and health status. Health status included smoking status (nonsmokers and smokers), drinking status (nondrinkers and drinkers) and chronic disease status (“never suffering from chronic diseases”, “only suffering from one chronic disease”, and "suffering from two or more chronic diseases"). The summary statistics for the control variables are shown in Table 1.

**Table 1:** The characteristic of the study population (N = 5471).

Variables	All participant (N = 5471)	HAP exposure time 3750		
		Free of HAP exposure (N = 1094)	Regular HAP exposure (N = 2845)	Sustained HAP exposure (N = 1422)
Pain (mean ± SD)	2.20 ± 1.28	1.93 ± 1.12	2.18 ± 1.28	2.44 ± 1.35
Depression (mean ± SD)	8.88 ± 6.71	7.04 ± 5.70	8.89 ± 6.71	10.31 ± 7.08
Disability (mean ± SD)	14.81 ± 4.00	13.83 ± 2.53	14.81 ± 3.98	15.56 ± 4.78
Sex (male, N, %)	2674 (48.88)	537 (49.17)	1446 (50.81)	637 (44.8)
Age (mean ± SD, years)	64.84 ± 8.69	64.16 ± 8.94	64.82 ± 8.82	65.42 ± 8.18
Residence (rural, N, %)	3425 (62.60)	323 (29.53)	1834 (64.47)	1207 (84.86)
Hukou (agricultural, N, %)	4209 (76.93)	539 (49.29)	2266 (79.64)	1326 (93.24)
Marital status (current married, N, %)	4070 (74.4)	823 (75.22)	2092 (73.54)	1073 (75.48)
Income (mean ± SD, yuan)	9.48 ± 1.03	10.07 ± 0.83	9.49 ± 0.99	9.01 ± 1.02
**Educational level (N, %)**				
No formal education	997 (17.87)	212 (19.37)	489 (17.19)	370 (26.03)
Primary school	2507 (45.84)	501 (45.76)	1316 (46.27)	717 (50.45)
Middle school	1195 (21.84)	232 (21.17)	657 (23.08)	230 (16.15)
High School or above	790 (14.44)	150 (13.7)	383 (13.46)	73 (7.37)
**Smoking status (N, %)**				
Non-smoker	3989 (72.91)	848 (77.54)	2072 (72.83)	987 (69.42)
Smoker	1482 (27.09)	246 (22.46)	772 (27.17)	435 (30.58)
**Drinking status (N, %)**				
Never	3640 (66.54)	668 (61.10)	1926 (67.69)	974 (68.52)
≥ 1 time/month	1403 (25.65)	302 (27.60)	716 (25.15)	357 (25.13)
< 1 time/month	427 (7.81)	124 (11.3)	294 (7.16)	90 (6.36)
**No. of chronic diseases (N, %)**				
0	794 (14.52)	167 (15.28)	419 (14.72)	192 (13.52)
1	1209 (22.11)	277 (25.29)	608 (21.37)	300 (21.09)
≥ 2	3467 (63.37)	650 (59.44)	1818 (63.91)	987 (69.42)

*HAP* household air pollution, *SD* standard deviation.

### Statistical analysis

The control variables, HAP, pain, depression and disability were described with mean, standard deviation (SD), or number (N) and percentage (%) as appropriate. To test the mediation role of pain, depression and HAP with disability, we first used Pearson product-moment correlation coefficient to preliminarily verified the hypothesis that pain, depression, and HAP are associated with disability. To ensure the accuracy of the results, and also to address the limitations of cross-sectional analysis that may lead to bias in evaluating mediation^63,79^. We ran alternative models to test the hypotheses that pain, depression, and HAP are associated with disability. All of the covariates were entered into the Logistic regression model, the Alternative Model 1 verified the association of HAP with disability, Alternative Model 2 and Alternative Model 3 for HAP with pain and depression, and Alternative Model 4 and Alternative Model 5 for pain and depression with disability, respectively. The Alternative Model 6 verified the association of pain and depression.

Second, after observing the association between the variables, a serial mediation model with four factors was applied to investigate whether the association between HAP and disability was mediated by pain and depression. Three mediation model tests were conducted successively, and these three models included two simple mediation pathways (the triangle pathways): (1) HAP → pain → disability; (2) HAP → depression → disability, and one serial mediation pathway (the quadrangle pathway): HAP → pain → depression → disability. Serial mediation model was analyzed using the PROCESS macro of SAS proposed by Preacher and Hayes.

We used the bootstrapping method with 5000 resamples of the data to test the significance of the indirect effects and the effects were reported with 95% confidence intervals (95% CIs). IBM SPSS Statistics 28.0 was used to perform all of the above analyses.

## Results

### Descriptive analysis

The basic characteristics of the included participants are described in Table 1*.* Overall, the mean (SD) age of the participants included in our study was 64.84 (8.69) years. Most of the participants (62.6%) had lived in rural areas. The participants who were exposed to HAP for more than 8 years more commonly lived in rural areas and had lower socioeconomic status than those who were exposed to HAP for less than 8 years. We also found that the individuals who were exposed to HAP for 1–8 years reported greater levels of pain, depression, and disability than those who were exposed to HAP for 0 years. The individuals who were exposed to HAP for 0 years had significantly lower levels of pain, depression, and disability than the average for all participants.

### Relationship between variables

To confirm our initial hypothesis, first, we examined the bivariate correlation between the key study variables (see Table 2). The middle-aged and older adults with higher levels of HAP exposure had higher levels of pain (r = 0.14, p < 0.01), depression (r = 0.17, p < 0.01), and disability (r = 0.15, p < 0.01). The middle-aged and older adults with higher levels of disability had higher levels of pain (r = 0.32, p < 0.01) and depression (r = 0.34, p < 0.01). Additionally, pain levels were significantly associated with depression (r = 0.39, p < 0.01). This result tentatively supported our hypothesis that correlations between each pair of the four factors, i.e., HAP, pain, depression, and disability was valid.

**Table 2:** Bivariate correlations (N = 5471).

Variable	Mean + SD	HAP	Pain	Depression	Disability
HAP	0.68 ± 1.06	1			
Pain	2.20 ± 1.28	0.14**	1		
Depression	8.88 ± 6.71	0.17**	0.39**	1	
Disability	14.81 ± 4.00	0.15**	0.32**	0.34**	1

*p < 0.05, **p < 0.01.

In the alternative models, all of the covariates were entered into the Alternative Model 1–6, as shown in Supplementary Table 2, the Alternative Model 1 verified that HAP was significantly associated with disability (B = 0.59, P < 0.01), Alternative Model 2 and Alternative Model 3 verified that HAP was negatively associated with pain (B = 0.16, P < 0.01) and depression (B = 0.74, P < 0.01), and Alternative Model 4 and Alternative Model 5 verified that pain (B = 0.84, P < 0.01) and depression (B = 0.18, P < 0.01) were negatively associated with disability, respectively. The Alternative Model 6 verified that pain was negatively associated with depression (B = 1.59, P < 0.01).

### Simple mediation model analyses of pain and depression

The mediating roles of pain and depression between HAP and disability were examined using two simple mediation models. Control variables included sex, age, residence, hukou, education level, marital status, income, chronic disease status, and smoking and drinking status. For the simple mediation model A (HAP → pain → disability), the analysis (see Table 3) revealed that HAP was positively related to disability (b = 0.59, p < 0.01) and pain (b = 0.16, p < 0.01). Pain was positively related to disability (b = 0.82, p < 0.01). Moreover, for the simple mediation model B (HAP → depression → disability), HAP was positively related to disability (b = 0.59, p < 0.01) and depression (b = 1.58, p < 0.01). Depression was positively related to disability (b = 0.18, p < 0.01). Detailed simple mediation analysis results are presented in Supplementary Table 3.

**Table 3:** Simplified simple mediation analysis results (N = 5471).

	Model 1	Model 2	Model 3	Model 4	Model 5	Model 6	Model 7
	Disability	Pain	Disability	Depression	Disability	Depression	Disability
Sex	0.17	0.44**	− 0.19	1.58**	− 0.11	0.89**	− 0.32*
age	0.072**	− 0.01*	0.08**	− 0.07**	0.08**	− 0.06**	0.09**
Residence	0.08	0.09	0.00	0.86**	− 0.08	0.72**	− 0.10
Hukou	0.06	0.04	0.03	0.40	− 0.01	0.34	− 0.02
Educational level	− 0.41**	− 0.12**	− 0.31**	− 0.87**	− 0.25**	− 0.68**	− 0.21**
Marital status	0.50**	0.05	0.46**	1.23**	0.28	1.15**	0.29*
Income	− 0.05	− 0.03	− 0.02	− 0.46**	0.04	− 0.42**	0.04
chronic diseases	0.82**	0.47**	0.43**	1.78**	0.50**	1.04**	0.28**
Smoking status	− 0.29	0.10	− 0.37*	0.58*	− 0.39*	0.58*	− 0.43**
Drinking status	− 0.43**	− 0.010*	− 0.35*	− 0.74**	− 0.30*	− 0.59**	− 0.27*
HAP	0.59**	0.16**	0.46**	1.58**	0.45**	0.49**	0.39**
PAIN	–	–	0.82**	–	–	1.57**	0.599**
Depression	–	–	–	–	0.18**	–	0.14**

Simple mediation A (HAP → pain → disability) include Model 1, Model 2 and Model 3; simple mediation B (HAP → depression → disability) include Model 1, Model 4 and Model 5; **p < 0.05, **p < 0.01; Serial Mediation (HAP → pain → depression → disability) include Model 2, Model 6, Model 1 and Model 7; *p < 0.05, **p < 0.01.

**Figure 2:**
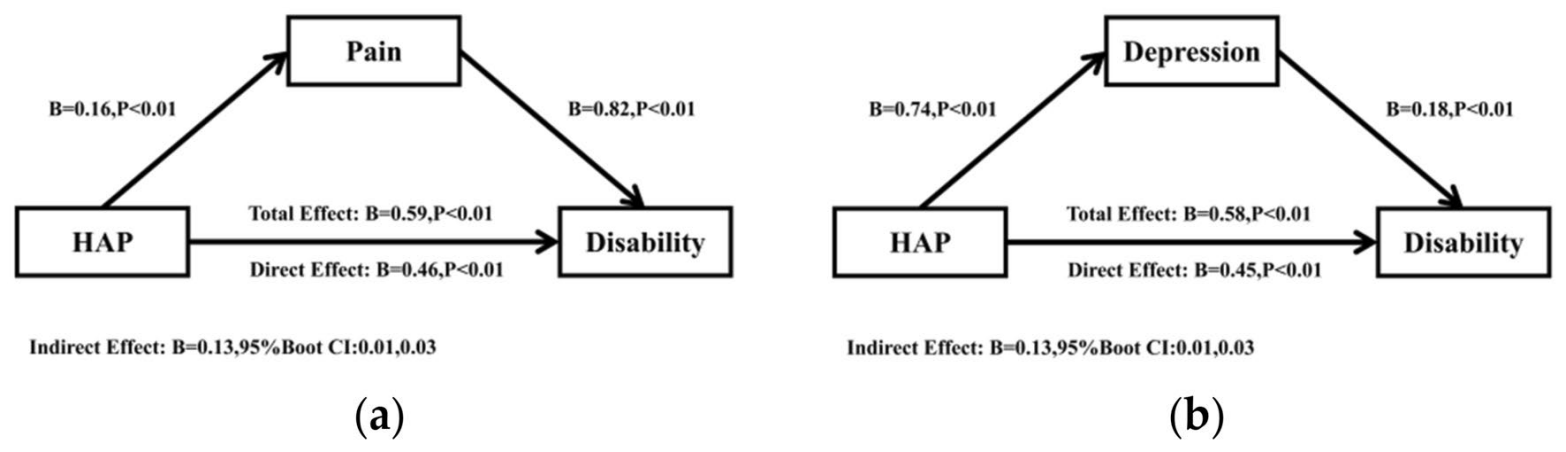
Simple mediation model of the association between HAP and Disability through Pain or Depression (N = 5471). (**a**) Simple mediation A (HAP → Pain → Disability); (**b**) simple mediation B (HAP → Depression → Disability).

Figure 2 shows the two simple mediation path models. The path coefficients illustrated that the direct effect of HAP on disability remained significant after incorporating the mediators of pain and depression. Thus, the association between HAP and disability could be realized through pain and depression.

To test whether the mediating effect of pain and depression was significant, two simple mediation analyses were performed with 5000 bootstrap samples. As shown in Table 4, pain and depression partially mediated the association between HAP and disability because the coefficients of the 95% CI for the paths did not include 0. The direct effect of HAP on disability was still significant after including pain (B = 0.82, 95% CI 0.73, 0.92). Pain significantly mediated the association between HAP and disability (indirect effect: B = 0.13, 95% CI 0.01, 0.03; Fig. 2A). The simple mediation model A showed that indirect effects explained 22.2% of the total effect of HAP on disability. Similarly, the direct effect of HAP on disability was still significant after including depression (B = 0.18, 95% CI 0.26, 0.65). Depression played a mediating role in the relationship between HAP and disability (indirect effect: B = 0.13, 95% CI 0.01, 0.03; Fig. 2B). The simple mediation model B showed that indirect effects explained 22.4% of the total effect of HAP on disability. Thus, pain and depression could be respectively used as intermediaries to influence the relationship between HAP and disability.

**Table 4:** Regression coefficients in the simple mediation model analysis (N = 5471).

Criterion	Predictors	R^2^	F	B	t	95% CI
**Simple mediating A(HAP → Pain → disability)**						
Pain	HAP	0.15	58.74	0.16	4.87	(0.09, 0.22)*
Disability	HAP	0.17	64.74	0.46	16.38	(0.26, 0.65)*
	Pain	–	–	0.82	4.57	(0.73, 0.92)*
**Simple mediating B(HAP → depression → disability)**						
Depression	HAP	0.14	54.89	0.74	4.34	(0.40, 1.07)*
Disability	HAP	0.19	72.87	0.45	18.84	(0.26, 0.65)*
	Depression	–	–	0.18	4.61	(0.16, 0.20)*

*Significant mediator (i.e., zero is not contained within the confidence intervals); sex, age, residence, Hukou, education level, marital status, income, chronic disease status, smoking status, and drinking status were analyzed as control variable.

### The serial mediation analyses of pain and depression

For the serial mediation model, the analysis results (see Tables 3 and 5) revealed that HAP had positive effects on pain (B = 0.16, p < 0.001, 95% CI 0.09, 0.22) and depression (B = 0.49, p < 0.001, 95% CI 0.17, 0.81). Pain was significantly related to depression (B = 1.57, p < 0.001, 95% CI 1.41, 1.73). In addition, both pain (B = 0.60, p < 0.001, 95% CI 0.50, 0.70) and depression (B = 0.14, p < 0.001, 95% CI 0.13, 0.16) were positively associated with disability. The association between HAP and disability persisted even after controlling for the effects of pain and depression (B = 0.39, p < 0.001, 95% CI 0.20, 0.58).

**Table 5:** Regression coefficients in the serial mediation analysis (N = 5471).

Criterion	Predictors	R^2^	F	B	t	95% CI
Pain	HAP	0.15	58.74	0.16	4.87	(0.09, 0.22)*
Depression	HAP	0.21	85.55	0.49	3.01	(0.17, 0.81)*
	Pain	–	–	1.57	19.08	(1.41, 1.73)*
Disability	HAP	0.22	80.17	0.39	3.97	(0.20, 0.58)*
	Pain	–	–	0.60	11.67	(0.50, 0.70)*
	Depression	–	–	0.14	14.83	(0.13, 0.16)*

*Significant mediator (i.e., zero is not contained within the confidence intervals); sex, age, education level, household income precipitant, marital status, chronic disease status, smoking and drinking status were analyzed as control variable.

**Figure 3:**
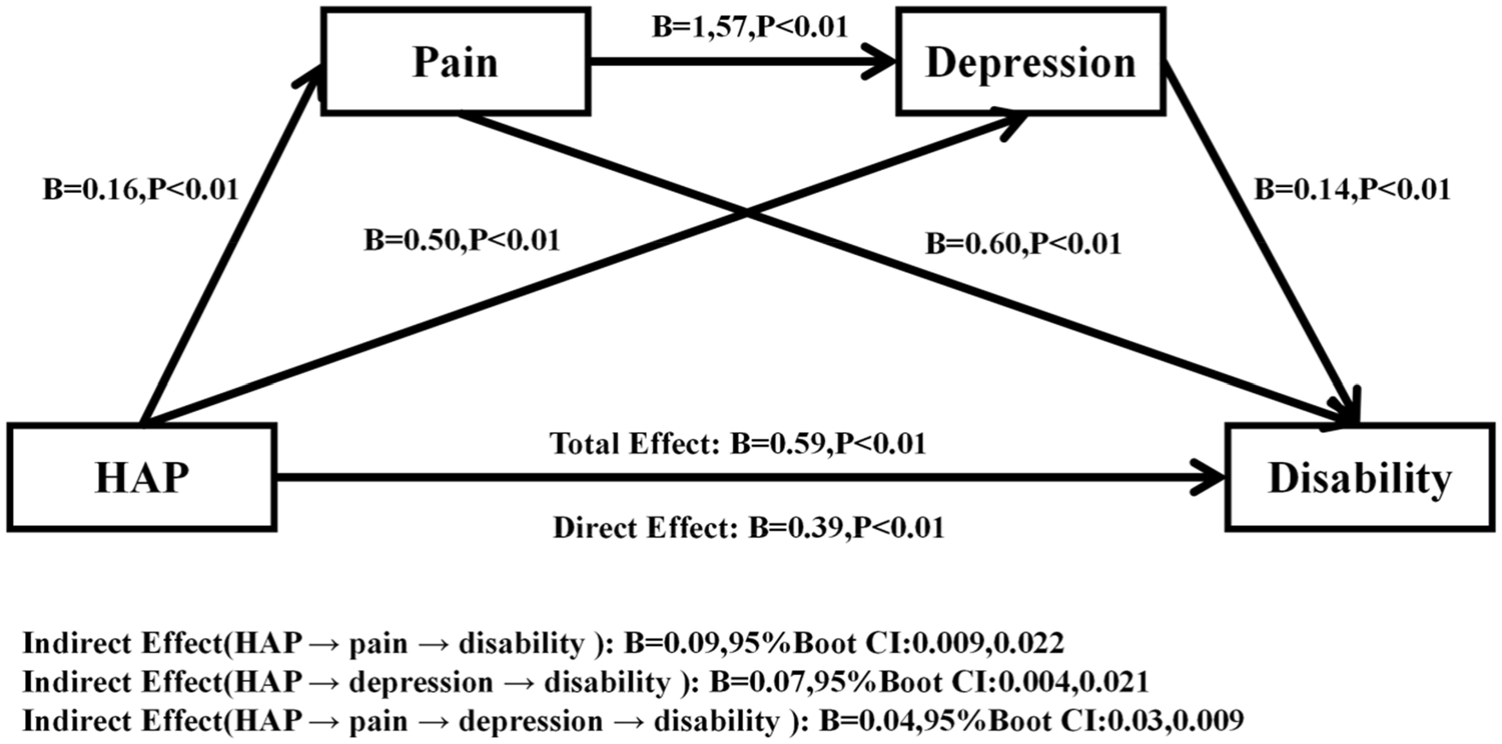
A serial mediation model of the association between HAP and disability through pain and depression (N = 5471). Path coefficients are shown. Mediation analyses were performed with 5000 bias-correct bootstrapped samples.

The serial mediation model of the association between HAP and disability through pain and depression is shown in Fig. 3. We tested the significance of the mediating effects of pain and depression using the bootstrap sampling test, the path effects of which were significant only when the 95% CI of the path coefficients did not include 0. We eventually identified a model of serial mediation, as shown in Fig. 3. When both pain and depression were simultaneously used as mediating variables in the whole model, three significant mediation pathways through which HAP indirectly impacts disability were found: (1) HAP → pain → disability (indirect effect: B = 0.09, 95% CI 0.009, 0.024), which accounted for 15.25% of the total effect; (2) HAP → depression → disability (indirect effect: B = 0.07, 95% CI 0.004, 0.021), accounting for 11.86% of the total effect; and (3) HAP → pain → depression → disability (indirect effect: B = 0.04, 95% CI 0.003, 0.009), which accounted for 6.78% of the total effect. Thus, all three mediating effect pathways exist.

## Discussion

This large cohort data study spanning 8 years (N = 5471) showed that exposure to HAP measured by use of solid fuels may be a risk factor for disability in middle-aged and older adults in China. We also found mediating mechanisms of pain and depression between exposure to HAP measured by use of solid fuels and disability in middle-aged and older adults. The logistic regression model showed a significant positive correlation between HAP and disability. This was consistent with the findings of a previous longitudinal study on middle-aged and older adults in China, which found that the risks of ADL disability and IADL disability increased by 39.9% and 71.0%, respectively, when they were exposed to HAP caused by solid fuels (coal, wood, or crop stubble) over a long period of time^14^. Middle-aged and older adults who have been exposed to HAP for a long time may have a higher risk of developing diseases in their lives, and disability often appears with the development of these diseases. Therefore, HAP is an important predictor of disability. Meanwhile, according to the serial mediation model, we found three pathways through which pain and depression were mediating variables that affected the relationship between exposure to HAP measured by use of solid fuels and disability. These findings suggested that HAP can indirectly affect disability through the respective and serial mediating roles of pain and depression. HAP caused by solid fuels can not only increase the risk of disability in middle-aged and older adults by inducing pain but also affect the risk of disability in middle-aged and older adults by altering their depression. More importantly, HAP can also induce changes in depression through pain, which in turn affects the risk of disability in middle-aged and older adults.

Our research showed that pain played a significant mediating role between HAP and disability. To a certain extent, it indicated that middle-aged and elderly people who have been exposed to HAP for a long time may suffer from disability due to pain. Previous studies have shown that HAP is related to the risk of pain in certain parts of the body (such as the chest and back, etc.)^26,64^, and pain has also been associated with the risk of disability. Buchman et al. found that in nondisabled community-dwelling older adults, the risk of disability increased with the number of reported musculoskeletal pain areas, and for each additional pain area, the risk of ADL disability increased by 20% and IADL disability increased by 10%^32^. On the basis of these findings, our results indicated that HAP could affect the level of pain in the elderly and thus affect the rate of disability among this population. This may be because when HAP causes pain in middle-aged and older adults, the subsequent stimulation of pain leads to a series of adverse cognitive processes, in which fear and illness resulting from pain eventually lead to disability^65^.

The findings also revealed that depression played a significant mediating role between HAP and disability. The findings showed that middle-aged and older adults who experienced long-term HAP exposure may also have an increased risk of disability due to depression. Previous studies have shown that elderly people are more likely to suffer from depression when they are exposed to HAP^12,28,66,67^. This may be because harmful particles in household air pollutants can interact with some human genes and change DNA methylation levels^68^ or induce metabolic changes consistent with the activation of the hypothalamus–pituitary–adrenal (HPA) axis^36,69^, which may influence the occurrence of depression or the degree of depression^70,71^. In addition, studies have found that depression is positively correlated with the risk of disability, and related studies have suggested that more attention should be given to individual psychological symptoms in the process of disability treatment^72^. Compared with physical symptoms, psychological symptoms contributed more to the reduction in the disability scores of Chinese patients with severe depression^72^. Our findings provide new evidence that explains the fact that when middle-aged and older adults suffer from psychological problems such as depression for a long time and are exposed to HAP for a long time, the risk of disability in middle-aged and older adults will be affected.

Finally, in this study, it was found that pain and depression had a serial mediating role between HAP and disability in middle-aged and older adults, which is consistent with previous research. Studies have shown that pain often causes more psychological distress, and this psychological distress often plays a mediating role between pain and disability^73,74^. Overall, middle-aged and older adults who have been exposed to HAP for a long time are more likely to develop disabilities through physical pain and severe depression. Therefore, in the future, we need to pay more attention to middle-aged and older adults who have been exposed to HAP for a long time to take intervention measures that reduce pain and psychological distress levels in these middle-aged and older adults.

Middle-aged and older adults who long-term exposure to HAP reported high levels of pain that caused the depression and ultimately increased the risk of disability. Prior research indicated that depression was seen as an adverse outcome of pain and had been shown to predict disability better than pain^47,75,76^. Those suffering from pain reported their prevalence of depression as high as 21%, 14% higher than the general population^77^. In many studies the complex relationship of pain and depression was confirmed and pain and depression can be seen as a components of a larger model^74^. Still, despite some evidence that depression played a significant mediating role between pain and frailty^78^ and other scholars have also shown that depression mediates the relationship between pain and functional limitations^73^, the toxicological mechanisms of this serial mediation model is not clear. Therefore, it is necessary to conduct more in-depth research to explore the toxicological mechanisms and causal relationships between HAP and disability.

Our research makes several contributions to this field. First, to the best of our knowledge, this was the first large cohort study to explore the mediating roles of pain and depression in the association between HAP and disability. Second, this study established the pathway of influence that showed that HAP affects pain, then pain affects depression, and finally, this leads to disability. Third, our research provides important information for governments that encourages the development and popularization of clean energy.

However, our study also had some limitations. First, we did not directly measure each individual's HAP level but evaluated it through questions concerning heating and cooking fuel use in the CHARLS questionnaire. Actual measurements of household air pollutants (such as PM2.5, carbon dioxide, nitrogen dioxide, etc.) will be necessary in the future to examine the actual air pollution-disability relationship. Second, the self-reported data concerning pain, depression, and disability may be less accurate than clinical interviews. Because the limitation of the questionnaire means that the disability may have existed much longer than depression (which is measured only in the last 7 days), we can not be too sure of the temporal relationship between depression and disability. Although our study used a cross-sectional study design, this is because previous research has shown that if the differences between longitudinal and cross-sectional analyses of mediation are small in practice, researchers may be justified in continuing to study mediation in cross-sectional designs, despite theoretical flaws^80^. But, longitudinal studies are needed in the future. Furthermore, a cross-sectional analysis may lead to bias in evaluating mediation, which is a longitudinal process^63^. In our study, we draw on previous research, the alternative models (association of depression with disability) are tested to overcome this limitation^79^.

Finally, we need to emphasize that there is a big difference between northern and southern China. In the south of China, most households do not have heating facilities, while in the north most households will install heating facilities. But whether north or south, all respondents are sure to know how to cook. Therefore, if it is simply distinguished from the use of only polluting fuels for cooking or heating, it will be affected regionally or territorially. And, due to CHARLS survey limitations, we cannot accurately distinguish the geographical location of respondents. So we can't overcome the influence of factors such as geography on the results. However in the future, it is very valuable to study the differences between the north and the south of China.

## Conclusions

This study provides new evidence for the association between HAP and disability and emphasizes the joint role of physical and psychological aspects in the mechanisms related to the effects of HAP on disability. Although there were some limitations in this study, we found that there were direct and indirect relationships between HAP and disability and that pain and depression can be used as intermediaries to influence the relationship between HAP and disability. Specifically, the serial mediation model we tested found that HAP measured by solid fuel use can increase the risk of disability in middle-aged and older adults through the serial mediation of pain and depression.

In conclusion, from this study, we gained a deeper understanding of the relationships between HAP and disability. From a clinical perspective, this study showed that improving the identification of pain and depression is very important for clinicians such that further intervention for middle-aged and older adults with disabilities can be provided. Finally, these conclusions support national strategies concerning the widespread use of clean fuels by citizens.

## Supplementary Information

Supplementary Information.

## Data Availability

The datasets used and analyzed during the current study are available and assessable from the China Health and Retirement Longitudinal Study (CHARLS, http://charls.pku.edu.cn/).
