# Supplementary material for: Effects of pain and depression on the relationship between household solid fuel use and disability among middle-aged and older adults
Source: Sci Rep. 2022 Dec 8;12:21270. doi: 10.1038/s41598-022-25825-8 (PMC9732289; doi:10.1038/s41598-022-25825-8)
Supplement: Supplementary file 1 — Supplementary Information. [file 41598_2022_25825_MOESM1_ESM.docx]

**Effects of pain and depression on the relationship between household solid fuel use and disability among middle-aged and older adults**

Zhihao Jia^1^, Yan Gao^1,^*, Liangyu Zhao^1^, Suyue Han^1^

1School of Physical Education, Shandong University, Jinan 250061, China

*corresponding. [gaoyanluck@sdu.edu.cn](mailto:gaoyanluck@sdu.edu.cn)

Correspondence: Address correspondence to Yan Gao, School of Physical Education, Shandong University. Full address: Jinan 250061, China. Email: [gaoyanluck@sdu.edu.cn](mailto:gaoyanluck@sdu.edu.cn)

**
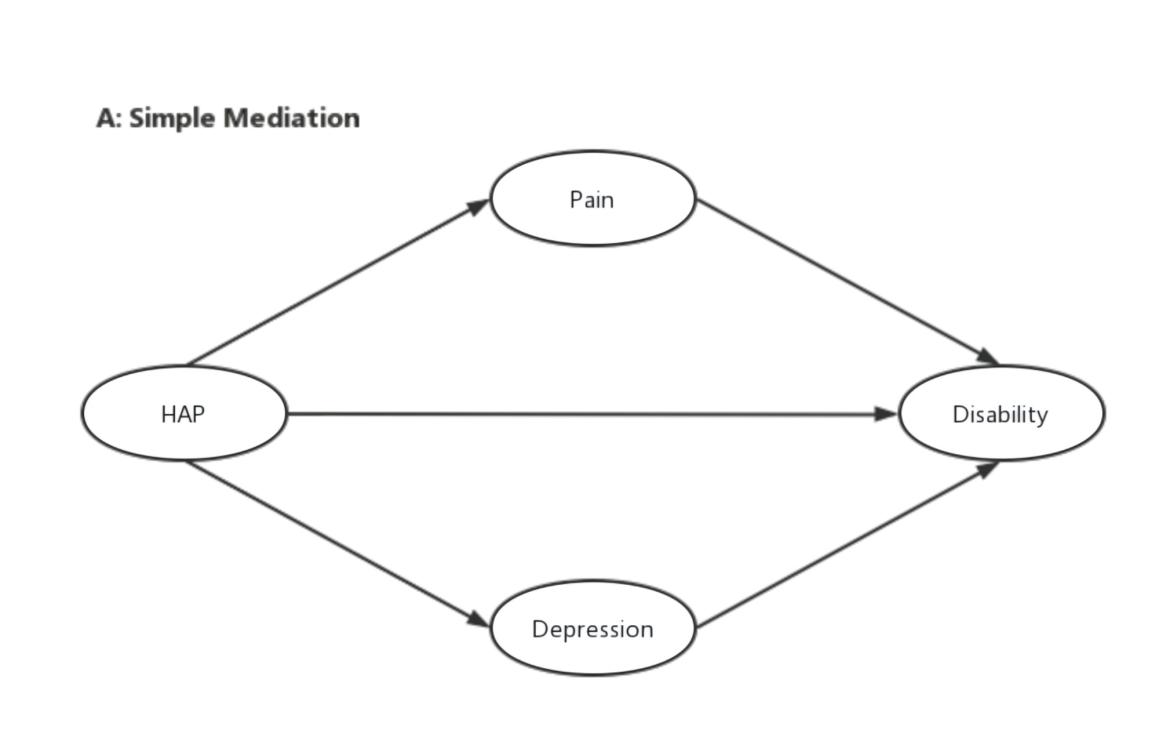
**

**Supplementary Figure 1 A.** Conceptual Models. Hypothesized simple mediation model linking HAP and disability through pain and depression as potential mediators; Note: HAP, household air pollution.


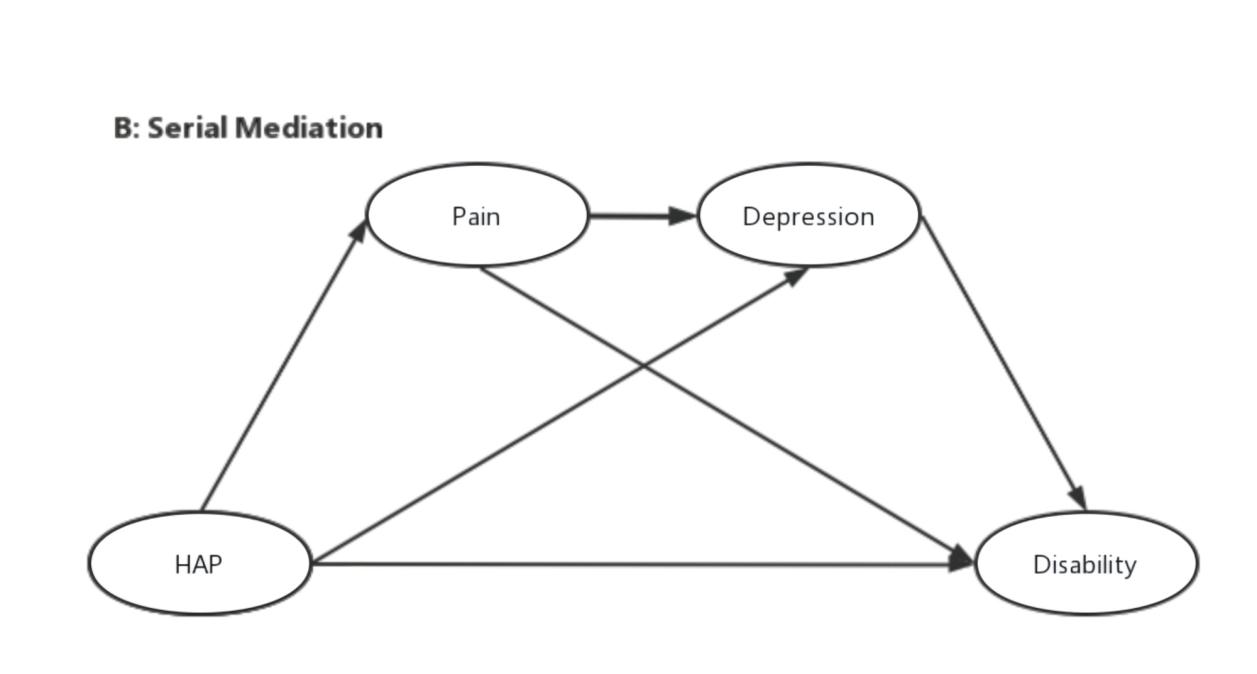


**Supplementary Figure 1 B.** Conceptual Models. Hypothesized serial mediation model linking HAP and disability through pain and depression as serial mediators. Note: HAP, household air pollution.

**Supplement Table 1** Questionnaire items for each variable in CHARLS

| **Variables** | **CHARLS questionnaire items** |
| --- | --- |
| HAP | 2018 CHARLS:I020; I021_W4; I022_W4  2018 CHARLS:I020; I021_W4; I022_W4  2018 CHARLS:I020; I021_W4; I022_W4 |
| PAIN | 2018 CHARLS: DA042 |
| Depression | 2018 CHARLS: DC009; DC010; DC011; DC012; DC013; DC014; DC015; DC016; DC017; DC018 |
| Sex | 2018 CHARLS: BA000_W2_3 |
| age | 2018 CHARLS: BA004_W3; BA005_W4; BA002 |
| Residence | 2018 CHARLS: BB001_W3 |
| Hukou | 2018 CHARLS: BC001_W3_1; BC001_W3_2; BC001_W3_3;BC002_W3; BC002_W3_1 |
| Educational level | 2018 CHARLS: BD001_W2_4 W; |
| Marital status | 2018 CHARLS: BE001 |
| Income | 2018 CHARLS: GE000_W4 |
| chronic diseases | 2018 CHARLS: DA007; DA007_1_; DA007_2_; DA007_3_; DA007_4_; DA007_5_; DA007_6_; DA007_7_; DA007_8_; DA007_9_; DA007_10_; DA007_11_; DA007_12_; DA007_13_; DA007_14_ |
| Smoking status | 2018 CHARLS: DA059; DA061; DA061_W4 |
| Drinking status | 2018 CHARLS: DA067 |

**Supplement Table 2** Correlations between the variables.

|  | **Model 1** | **Model 2** | **Model 3** | **Model 4** | **Model 5** | **Model 6** |
| --- | --- | --- | --- | --- | --- | --- |
|  | Disability | Pain | Depression | Disability | Disability | Depression |
| Sex | 0.171 | 0.443** | 1.580** | -0.212 | -0.125 | 0.866** |
| age | 0.072** | -0.007* | -0.068** | 0.079** | 0.085** | -0.057** |
| Residence | 0.078 | 0.092 | 0.860** | 0.146 | 0.068 | 0.870** |
| Hukou | 0.06 | 0.036 | 0.395 | 0.124 | 0.082 | 0.44 |
| Educational level | -0.405** | -0.121** | -0.870** | -0.328** | -0.273** | -0.705** |
| Marital status | 0.500** | 0.052 | 1.232** | 0.412** | 0.232 | 1.102** |
| Income | -0.046 | -0.029 | -0.464** | -0.09 | -0.03 | -0.491** |
| chronic diseases | 0.816** | 0.469** | 1.775** | 0.437** | 0.510** | 1.047** |
| Smoking status | -0.288 | 0.098 | 0.581* | -0.351* | -0.374* | 0.446 |
| Drinking status | -0.430** | -0.095* | -0.736** | -0.369** | -0.316* | -0.605** |
| HAP | 0.585** | 0.157** | 0.738** | —— | —— | —— |
| PAIN | —— | —— | —— | 0.842** | —— | 1.590** |
| Depression | —— | —— | —— | —— | 0.181** | —— |

* p<0.05 ** p<0.01

**Supplement Table 3** Detailed simple mediation analysis results.

|  | **simple mediation A** | | | **simple mediation B** | | | **Serial Mediation** | | | |
| --- | --- | --- | --- | --- | --- | --- | --- | --- | --- | --- |
|  | Disability | Pain | Disability | Disability | Depression | Disability | Pain | Depression | Disability | Disability |
| Sex | 0.171 | 0.443** | -0.194 | 0.171 | 1.580** | -0.11 | 0.443** | 0.885** | 0.171 | -0.321* |
| age | 0.072** | -0.007* | 0.078** | 0.072** | -0.068** | 0.084** | -0.007* | -0.058** | 0.072** | 0.086** |
| Residence | 0.078 | 0.092 | 0.002 | 0.078 | 0.860** | -0.076 | 0.092 | 0.715** | 0.078 | -0.102 |
| Hukou | 0.06 | 0.036 | 0.031 | 0.06 | 0.395 | -0.01 | 0.036 | 0.339 | 0.06 | -0.018 |
| Educational level | -0.405** | -0.121** | -0.305** | -0.405** | -0.870** | -0.250** | -0.121** | -0.680** | -0.405** | -0.207** |
| Marital status | 0.500** | 0.052 | 0.458** | 0.500** | 1.232** | 0.281 | 0.052 | 1.151** | 0.500** | 0.292* |
| Income | -0.046 | -0.029 | -0.022 | -0.046 | -0.464** | 0.036 | -0.029 | -0.418** | -0.046 | 0.038 |
| chronic diseases | 0.816** | 0.469** | 0.429** | 0.816** | 1.775** | 0.499** | 0.469** | 1.039** | 0.816** | 0.280** |
| Smoking status | -0.288 | 0.098 | -0.369* | -0.288 | 0.581* | -0.392* | 0.098 | 0.427 | -0.288 | -0.430** |
| Drinking status | -0.430** | -0.095* | -0.352* | -0.430** | -0.736** | -0.299* | -0.095* | -0.587** | -0.430** | -0.267* |
| HAP | 0.585** | 0.157** | 0.455** | 0.585** | 0.738** | 0.454** | 0.157** | 0.490** | 0.585** | 0.385** |
| PAIN | —— | —— | 0.824** | —— | —— | —— | —— | 1.570** | —— | 0.598** |
| Depression | —— | —— | —— | —— | —— | 0.178** | —— | —— | —— | 0.144** |

* p<0.05 ** p<0.01
